# Supplementary material for: Cytoplasmic retention and degradation of a mitotic inducer enable plant infection by a pathogenic fungus
Source: eLife. 2019 Oct 17;8:e48943. doi: 10.7554/eLife.48943 (PMC6887120; doi:10.7554/eLife.48943)
Supplement: Supplementary file 1. [file elife-48943-supp1.doc]

**Supplementary File 1.** *U. maydis* strains used in this study

| **Strain** | **Relevant genotype** | **Source** |
| --- | --- | --- |
| FB1 | *a1, b1* |  |
| FB2 | *a2, b2* | [Banuett and Herskowitz, 1989](#_ENREF_3)) |
| FB1Pcrg:fuz7DD | *a1, b1, ip::P_crg1_:fuz7^DD^* |  |
| UMS2 | *a1, b1, ip::P_crg1_:fuz7^DD^,cut11-Cherry-P_tef1_:NLS-GFP-Phleo^R^* | This work |
| UMS3 | *a1, b1, ip::P_crg1_:fuz7^DD^,cut11-Cherry-P_tef1_:NLS-GFP-Phleo^R^, IG::P_crg1_:cdk1-3myc-Hyg^R^* | This work |
| UMS5 | *a1, b1, ip::P_crg1_:fuz7^DD^,cut11-Cherry-P_tef1_:NLS-GFP-Phleo^R^, IG::P_crg1_:cdk1^AF^-3myc-Hyg^R^* | This work |
| UMS15 | *a1, b1, ip::P_crg1_:fuz7^DD^,cut11-Cherry-P_tef1_:NLS-GFP-Phleo^R^, wee1^nar1^-Hyg^R^* | This work |
| UMPB12 | *a1, b1, clb2-3HA-Hyg^R^* | This work |
| UMPB15 | *a1, b1, ip::P_crg1_:fuz7^DD^, clb2-3HA-Hyg^R^* | This work |
| UMS195 | *a1, b1, cdc25-3HA-Hyg^R^* | This work |
| UMPB16 | *a1, b1, ip::P_crg1_:fuz7^DD^, cdc25-3HA-Hyg^R^* | This work |
| UMS200 | *a1, b1, wee1-3HA-Hyg^R^* | This work |
| UMPB14 | *a1, b1, ip::P_crg1_:fuz7^DD^, wee1-3HA-Hyg^R^* | This work |
| UMPB109 | *a1, b1, ip::P_crg1_:fuz7^DD^, cut11-Cherry-P_tef1_:NLS-GFP-Phleo^R^, IG::P_crg1_:cdc25-3HA- Hyg^R^* | This work |
| UMS125 | *a1, b1, ip::P_crg1_:fuz7^DD^, cut11-Cherry-P_tef1_:NLS-GFP-Phleo^R^, pcl12*∆*:: G418^R^* | This work |
| UMS136 | *a1, b1, ip::P_crg1_:fuz7^DD^, cut11-Cherry-P_tef1_:NLS-GFP-Phleo^R^, crk1*∆*:: Hyg^R^* | This work |
| UMPB18 | *a1, b1, ip::P_crg1_:fuz7^DD^, cdc25-3HA-Hyg^R^, pcl12*∆*:: G418^R^* | This work |
| UMPB25 | *a1, b1, ip::P_crg1_:fuz7^DD^, cdc25-3HA-Hyg^R^, crk1*∆*:: G418^R^* | This work |
| UMS176 | *a1, b1, ip::P_crg1_:pcl12, cut11-Cherry-P_tef1_:NLS-GFP-Phleo^R^* | This work |
| UMS177 | *a1, b1, ip::P_crg1_:pcl12, cut11-Cherry-P_tef1_:NLS-GFP-Phleo^R^, crk1*∆*:: Hyg^R^* | This work |
| UMS179 | *a1, b1, ip::P_crg1_:pcl12, cut11-Cherry-P_tef1_:NLS-GFP-Phleo^R^, crk1^KD^- Hyg^R^* | This work |
| UMS243 | *a1, b1, ip::P_crg1_:pcl12, cut11-Cherry-P_tef1_:NLS-GFP-Phleo^R^, fuz7*∆*:: Hyg^R^* | This work |
| UMS244 | *a1, b1, ip::P_crg1_:pcl12, cut11-Cherry-P_tef1_:NLS-GFP-Phleo^R^, kpp2*∆*:: Hyg^R^* | This work |
| UMS245 | *a1, b1, ip::P_crg1_:pcl12, cut11-Cherry-P_tef1_:NLS-GFP-Phleo^R^, crk1^AEF^- Hyg^R^* | This work |
| UMS246 | *a1, b1, ip::P_crg1_:pcl12, cut11-Cherry-P_tef1_:NLS-GFP-Phleo^R^, crk1^AAA^- Hyg^R^* | This work |
| UMS184 | *a1, b1, ip::P_crg1_: fuz7^DD^, cut11-Cherry-P_tef1_:NLS-GFP-Phleo^R^, crk1^AEF^- Hyg^R^* | This work |
| UMS185 | *a1, b1, ip::P_crg1_: fuz7^DD^, cut11-Cherry-P_tef1_:NLS-GFP-Phleo^R^, crk1^AAA^- Hyg^R^* | This work |
| UMPB111 | *a1, b1, ip::P_crg1_:pcl12, cut11-Cherry-Phleo^R^, GFP-cdc25* | This work |
| UMPB112 | *a1, b1, ip::P_crg1_:pcl12, cut11-Cherry-Phleo^R^, GFP-cdc25, crk1*∆*:: Hyg^R^* | This work |
| UMPB114 | *a1, b1, kap123-3HA-Hyg^R.^* | This work |
| UMPB115 | *a1, b1, ip::P_crg1_: fuz7^DD^, kap123-3HA-Hyg^R.^* | This work |
| UMPB116 | *a1, b1, ip::P_crg1_: pcl12, kap123-3HA-Hyg^R.^* | This work |
| UMPB130 | *a1, b1, kap123-GFP-G418^R^, cdc25-3HA-Hyg^R^* | This work |
| UMPB131 | *a1, b1, ip::P_crg1_: pcl12, kap123-GFP-G418^R^, cdc25-3HA-Hyg^R^* | This work |
| UMPB132 | *a1, b1, ip::P_crg1_: pcl12, kap123-GFP-G418^R^, cdc25-3HA-Hyg^R^, crk1*∆*:: Nat^R^* | This work |
| UMPB135 | *a1, b1, ip::P_crg1_: wee1, kap123-GFP-G418^R^, cdc25-3HA-Hyg^R^* | This work |
| UMPB143 | *a1, b1, ip::P_crg1_: fuz7^DD^, kap123^T867A^-3HA-Hyg^R.^* | This work |
| UMPB146 | *a1, b1, ip::P_crg1_:pcl12, kap123^T867A^-3HA-Hyg^R.^* | This work |
| UMPB148 | *a1, b1, ip::P_crg1_: fuz7^DD^, cut11-Cherry-P_tef1_:NLS-GFP-Phleo^R^, kap123-3HA-Hyg^R^* | This work |
| UMPB149 | *a1, b1, ip::P_crg1_: fuz7^DD^, cut11-Cherry-P_tef1_:NLS-GFP-Phleo^R^, kap123^T867A^-3HA-Hyg^R^* | This work |
| UMPB150 | *a1, b1, ip::P_crg1_: pcl12, cut11-Cherry-P_tef1_:NLS-GFP-Phleo^R^, kap123-3HA-Hyg^R^* | This work |
| UMPB151 | *a1, b1, ip::P_crg1_: pcl12, cut11-Cherry-P_tef1_:NLS-GFP-Phleo^R^, kap123^T867A^-3HA-Hyg^R^* | This work |
| UMPB156 | *a1, b1, ip::P_crg1_: pcl12, kap123^T867A^-GFP-G418^R^, cdc25-3HA-Hyg^R^* | This work |
| UMPB158 | *a1, b1, ip::P_crg1_:pcl12, cut11-Cherry-Phleo^R^, GFP-cdc25, kap123^T867A^-3HA-Hyg^R^* | This work |
| UMPB159 | *a1, b1, kap123^T867A^-3HA-Hyg^R.^* | This work |
| UMPB160 | *a1, b1, cut11-Cherry-Phleo^R^, GFP-cdc25, kap123-3HA-Hyg^R^* | This work |
| UMPB161 | *a1, b1, cut11-Cherry-Phleo^R^, GFP-cdc25, kap123^T867A^-3HA-Hyg^R^* | This work |
| UMPB170 | *a1, b1, kap123-frt .* | This work |
| UMPB171 | *a2, b2, kap123-frt .* | This work |
| UMPB172 | *a1, b1, kap123^T867A^-frt .* | This work |
| UMPB173 | *a2, b2, kap123^T867A^-frt .* | This work |
| UMPB174 | *a1, b1, kap123-frt, ip:: P_dik6_:NLS-GFP* | This work |
| UMPB175 | *a1, b1, kap123^T867A^-frt, ip:: P_dik6_:NLS-GFP* | This work |
| UMPB179 | *a1, b1, ip::P_crg1_: fuz7^DD^, cdc25-3HA-Hyg^R^, kap123^T867A^-frt* | This work |
| UMPB162 | *a1, b1, ip::P_crg1_:fuz7^DD^, IG::P_crg1_:Pcl12-Phleo^R^, cdc25-3HA-Hyg^R^* | This work |
| UMPB163 | *a1, b1, ip::P_crg1_:fuz7^DD^, IG::P_crg1_:Pcl12-Phleo^R^, cdc25-3HA-Hyg^R^, kpp2*∆*:: G418^R^* | This work |
| UMPB164 | *a1, b1, IG::P_crg1_:Pcl12-Phleo^R^, cdc25-3HA-Hyg^R^, kpp2^K50R^-GFP-G418^R^* | This work |
| UMPB165 | *a1, b1, ip::P_crg1_:fuz7^DD^, IG::P_crg1_:Pcl12-Phleo^R^, cdc25-3HA-Hyg^R^, , kpp2^K50R^-GFP-G418^R^* | This work |
| UMPB166 | *a1, b1, ip::P_crg1_:fuz7^DD^, IG::P_crg1_:Pcl12-Phleo^R^, cdc25-3HA-Hyg^R^, kpp2^K50R^-GFP-G418^R^ , kap123^T867A^-frt* | This work |
| UMPB168 | *a1, b1, ip::P_crg1_:fuz7^DD^, cdc25*∆1-270*-3HA-Hyg^R^* | This work |
| UMPB169 | *a1, b1, ip::P_crg1_:fuz7^DD^, cdc25*^AAA^*-3HA-Hyg^R^* | This work |
| UMPB180 | *a1, b1, ip::P_crg1_:fuz7^DD^, cut11-Cherry-P_tef1_:NLS-GFP-Phleo^R^, cdc25*∆1-270*-3HA-Hyg^R^* | This work |
| UMPB181 | *a1, b1, ip::P_crg1_:fuz7^DD^, cut11-Cherry-P_tef1_:NLS-GFP-Phleo^R^, cdc25*^AAA^*-3HA-Hyg^R^* | This work |
| UMPB186 | *a1, b1, cdc25^AAA.^* | This work |
| UMPB187 | *a2, b2, cdc25^AAA^* | This work |
| UMPB188 | *a1, b1, cdc25, ip:: P_dik6_:NLS-GFP.* | This work |
| UMPB189 | *a1, b1, cdc25^AAA^, ip:: P_dik6_:NLS-GFP* | This work |
| SG200 | *a1, mfa2, bW2, bE1* |  |
| UMPB192 | *a1, mfa2, bW2, bE1, cdc25^AAA^* | This work |
| UMP121 | *a2, bW2^nar1^,* *bE1^nar1^, ip:: P_dik6_:NLS-GFP.* |  |
| UMPB190 | *a2, bW2^nar1^,* *bE1^nar1^, cdc25^AAA^-frt, ip:: P_dik6_:NLS-GFP.* | This work |
| UMS26 | *a1, b1, ip::P_crg1_:fuz7^DD^, kpp2*∆*:: Hyg^R^* | This work |
| UMS28 | *a1, b1, ip::P_crg1_:fuz7^DD^, prf1*∆*:: Hyg^R^* | This work |
| UMS51 | *a1, b1, ip::P_crg1_:fuz7^DD^,cut11-Cherry-P_tef1_:NLS-GFP-Phleo^R^, chk1*∆*:: Hyg^R^* | This work |
| UMS73 | *a1, b1, ip::P_crg1_:fuz7^DD^,cut11-Cherry-P_tef1_:NLS-GFP-Phleo^R^, hsl1^tef1^-Nat^R^* | This work |
| UMS175 | *a1, b1, ip::P_crg1_:fuz7^DD^,cut11-Cherry-P_tef1_:NLS-GFP-Phleo^R^, chk1*∆*:: Hyg^R^, hsl1^tef1^-Nat^R^* | This work |
| UMS203 | *a1, b1, ip::P_crg1_:fuz7^DD^,cut11-Cherry-P_tef1_:NLS-GFP-Phleo^R^, cdk5^ts^-Nat^R^* | This work |
| UMPB62 | *a1, b1, ip::P_crg1_:fuz7^DD^, cdc25-3HA-Hyg^R^, cdk5^ts^-Nat^R^* | This work |
| UMPB33 | *a1, b1, ip::P_crg1_:fuz7^DD^, pcl12-GFP-Hyg^R^* | This work |
| UMS144 | *a1, b1, ip::P_crg1_:fuz7^DD^, cut11-Cherry-P_tef1_:NLS-GFP-Phleo^R^, pcl12*∆*:: G418^R^, crk1*∆*:: Hyg^R^* | This work |
| UMS198 | *a1, b1, ip::P_crg1_:pcl12,cut11-Cherry-P_tef1_:NLS-GFP-Phleo^R^, IG::P_crg1_:cdk1-3myc-Hyg^R^* | This work |
| UMS199 | *a1, b1, ip::P_crg1_:pcl12,cut11-Cherry-P_tef1_:NLS-GFP-Phleo^R^, IG::P_crg1_:cdk1^AF^-3myc-Hyg^R^* | This work |
| UMP276 | *a1, b1, ip::P_crg1_:pcl12,cdc25-3HA-Hyg^R^* | This work |
| UMP299 | *a1, b1, srp1^nar1^-Hyg^R^* | This work |
| UMP300 | *a1, b1, kap123^nar1^-Hyg^R^* | This work |
| UMP301 | *a1, b1, GFP-cdc25* | This work |
| UMP302 | *a1, b1, srp1^nar1^-Hyg^R^, GFP-cdc25* | This work |
| UMP303 | *a1, b1, kap123^nar1^-Hyg^R^, GFP-cdc25* | This work |
| UMPB120 | *a1, b1, ip::P_crg1_: fuz7^DD^, kap123-3HA-Hyg^R^, crk1*∆*:: Nat^R^* | This work |
| UMPB121 | *a1, b1, ip::P_crg1_: fuz7^DD^, kap123-3HA-Hyg^R^, pcl12*∆*:: G418^R^* | This work |
| UMPB122 | *a1, b1, ip::P_crg1_: fuz7^DD^, kap123-3HA-Hyg^R^, kpp2*∆*:: G418^R^* | This work |
| UMPB125 | *a1, b1, ip::P_crg1_: pcl12, kap123-3HA-Hyg^R^, crk1*∆*:: Nat^R^* | This work |
| UMPB126 | *a1, b1, ip::P_crg1_: pcl12, kap123-3HA-Hyg^R^, fuz7*∆*:: G418^R^* | This work |
| UMPB127 | *a1, b1, ip::P_crg1_: pcl12, kap123-3HA-Hyg^R^, kpp2*∆*::G418^R^* | This work |
| UMPB140 | *a1, b1, ip::P_crg1_: fuz7^DD^, kap123^S52A^-3HA-Hyg^R.^* | This work |
| UMPB141 | *a1, b1, ip::P_crg1_: fuz7^DD^, kap123^T595A^-3HA-Hyg^R.^* | This work |
| UMPB142 | *a1, b1, ip::P_crg1_: fuz7^DD^, kap123^S809A^-3HA-Hyg^R.^* | This work |
| UMPB144 | *a1, b1, ip::P_crg1_: fuz7^DD^, kap123^S994A^-3HA-Hyg^R.^* | This work |
| UMPB198 | *a2, bW2^nar1^,* *bE1^nar1^, kap123^T867A^-3HA-Hyg^R^, ip:: P_dik6_:NLS-GFP.* | This work |
| UMP320 | *a1, b1, ip::P_crg1_:pcl12, cut11-Cherry-Phleo^R^, GFP- cdc25*∆1-270 | This work |
| UMP321 | *a1, b1, ip::P_crg1_:pcl12, cut11-Cherry-Phleo^R^, GFP-cdc25*^AAA^ | This work |

Banuett, F., and Herskowitz, I. (1989). Different a alleles of Ustilago maydis are necessary for maintenance of filamentous growth but not for meiosis. Proc Natl Acad Sci U S A *86*, 5878-5882.

Bolker, M., Genin, S., Lehmler, C., and Kahmann, R. (1995). Genetic regulation of mating and dimorphism in Ustilago maydis. Can J Bot *73*, S320-S325.

Mielnichuk, N., Sgarlata, C., and Perez-Martin, J. (2009). A role for the DNA-damage checkpoint kinase Chk1 in the virulence program of the fungus Ustilago maydis. J Cell Sci *122*, 4130-4140.

Muller, P., Weinzierl, G., Brachmann, A., Feldbrugge, M., and Kahmann, R. (2003). Mating and pathogenic development of the Smut fungus Ustilago maydis are regulated by one mitogen-activated protein kinase cascade. Eukaryotic cell *2*, 1187-1199.
